# Nonlinearly-enhanced energy transport in many dimensional quantum chaos

**DOI:** 10.1038/srep02359

**Published:** 2013-08-05

**Authors:** D. S. Brambila, A. Fratalocchi

**Affiliations:** 1 PRIMALIGHT, Faculty of Electrical Engineering; Applied Mathematics and Computational Science, King Abdullah University of Science and Technology (KAUST), Thuwal 23955-6900, Saudi Arabia; 2 Max-Born Institute, Max-Born-Straβe 2 A, 12489, Berlin, Germany

## Abstract

By employing a nonlinear quantum kicked rotor model, we investigate the transport of energy in multidimensional quantum chaos. This problem has profound implications in many fields of science ranging from Anderson localization to time reversal of classical and quantum waves. We begin our analysis with a series of parallel numerical simulations, whose results show an unexpected and anomalous behavior. We tackle the problem by a fully analytical approach characterized by Lie groups and solitons theory, demonstrating the existence of a universal, nonlinearly-enhanced diffusion of the energy in the system, which is entirely sustained by soliton waves. Numerical simulations, performed with different models, show a perfect agreement with universal predictions. A realistic experiment is discussed in two dimensional dipolar Bose-Einstein-Condensates (BEC). Besides the obvious implications at the fundamental level, our results show that solitons can form the building block for the realization of new systems for the enhanced transport of matter.

Anderson localization is a fundamental concept that, originally introduced in solid-state physics to describe conduction-insulator transitions in disordered crystals, has permeated several research areas and has become the subject of great research interest^1^,^2^,^3^,^4^,^5^,^6^,^7^,^8^,^9^,^10^,^11^. Theories and subsequent experiments demonstrated that disorder favors the formation of spatially localized states, which sustain diffusion breakdown and exponentially attenuated transmission in random media^1^. Although many properties of wave localization are now well understood, several fundamental questions remains. Perhaps one of the most intriguing problem is related to the transport of energy. Intuitively, one can expect that disorder –by favoring exponentially localized stated– arrests in general any propagation inside a noncrystalline medium. However, the interplay between localization and disorder is nontrivial^5^,^12^,^13^ and under specific conditions randomness can significantly enhance energy transport. In linear regime, in particular, it has been observed that quasi-crystals with multifractal eigenstates and/or material systems with temporal fluctuations of the potential (or refractive index), lead to anomalous diffusion in the phase space^14^,^15^,^16^,^17^,^18^. This originates counterintuitive dynamics including ultralow conductivities^14^, as well as the formation of mobility edges even in one dimensional systems^17^. All these studies focused on linear materials and they did not investigate the role of nonlinearity for further controlling the transport of energy in many dimensions.

In the context of quantum localization, the problem of energy transport has stirred a conspicuous interest as well. In this area, quantum-classical correspondences mediated by Anderson localization possess many implications in the irreversible behavior of time reversible systems, which are at the basis of a long standing physical debate –i.e., the Loschmidt paradox^19^. Started a few centuries ago as a controversy between Boltzmann and Loschmidt, this famous paradox is still the matter of intense research in the scientific community^20^,^21^,^22^,^23^,^24^,^25^,^26^. The Loschmidt paradox deals with the origin of the irreversible behavior of time reversible systems that, according to the law of classical mechanics, should not manifest irreversible entropy growth as conversely predicted by the second law of thermodynamics. Time reversibility, in fact, guarantees that for every orbit that leads to an entropy increase there exists –with the same probability– a time-reversed path that generates the same entropy change but with an opposite sign^19^. Recent experiments might suggest that a possible solution of this paradox can be formulated in terms of deterministic chaos^23^. According to this interpretation, time reversibility is possible only at the quantum level, where Anderson localization breaks diffusive transport and suppresses the mixing ability of chaos, as discussed in one dimension by a series of papers^21^,^22^,^27^. The possibility to exploit quantum time reversal is at the basis of several nontrivial dynamics including the quantum-echo effect^21^,^22^,^26^. However, when more dimensions are considered, numerical simulations predict that ergodicity is fully restored and diffusive transport settles is again, thus re-establishing the classical features of chaos and preventing quantum time reversal and its associated dynamics^22^. Nevertheless, theoretical work reported to date considered only noninteracting systems, characterizesd by linear equations of motion. The Loschmidt paradox, conversely, involved the use of interacting atoms, whose interplay in the mean field regime is accounted by short and/or long ranged nonlinear responses^28^,^29^,^30^. As pointed out in the literature^21^, atoms interactions are of crucial importance in quantum localization and diffusion. A key question in this problem lies in understanding how nonlinearity affects the transport of energy in many dimensions. In one dimensional quantum chaotic systems, pioneering numerical experiments of Casati *et al.*^27^ reported that nonlinearity maintains dynamical localization effects. This conforms to the intuitive idea that a nonlinear response, due to its localization properties, works together Anderson effects to suppress mixing dynamics. Recent theoretical work performed on *d*-dimensional disordered lattices show that at any finite nonlinearity there exist a finite probability for the observation of Anderson localization effects^31^. In this scenario, nontrivial effects are expected to occur on the energy transport, due to the rich interplay between localization and nonlinearity, as well as by the additional degrees of freedom that can interact in the dynamics.

In this Article, we theoretically investigate this problem by employing both numerical simulations and analytic techniques. To pursue a general theory, we here consider the following two dimensional model: 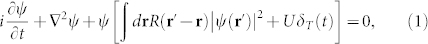
with **r** = (*x*, *y*), ∇^2^ = *∂*^2^/*∂x*^2^ + *∂*^2^/*∂y*^2^, *δ_T_* = Σ*_n_ δ*(*t* − *nT*) a periodic delta-function of period *T*, *R* a general nonlinear response and 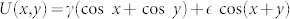 a two dimensional periodic potential with strength defined by 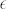 and *γ*. Equation (1) defines a two dimensional, nonlinear quantum kicked rotor: for *R* = 0 it reduces to the linear quantum kicked rotator^22^ while for *U* = 0 it corresponds to the 2D nonlinear Schrödinger equation (NLS), which represents a universal model of nonlinear waves in dispersive media^30^. In one dimension, conversely, Eq. (1) generalizes the nonlinear model investigated in^27^ with classical chaos parameter *K* = 2*γT*. Despite its deterministic nature, Eq. (1) can be precisely mapped to the Anderson model with a random potential^11^,^32^,^33^, and therefore furnishes a fundamental model for studying energy transport and dynamical localization^34^,^35^ in random systems.

This article is organized as follows. We begin by a series of experiments studying the diffusion of energy by integrating Eq. (1) in time with a parallel algorithm. We interpreted the results by an analytic theory based on Lie symmetry groups and soliton waves, which predicts a universal enhanced diffusion process, which is entirely sustained by nonlinearity. Analytical results are validated against numerical simulations, showing a perfect agreement with our predictions. We finally discuss the realization of a possible experiments in a multidimensional dipolar BEC, showing the feasibility of our ideas in a realistic setting.

## Results

### A first numerical experiment

**Figure 1:**
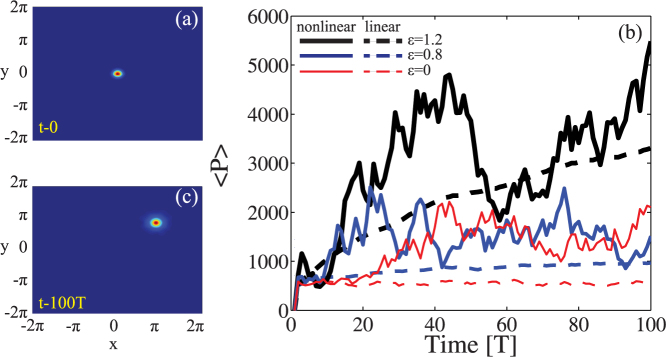
(a)–(b) spatial density |*ψ*|^2^ distribution at (a) *t* = 0 and at (b) *t* = 100*T*; (c) momentum diffusion 〈*P*〉 versus time in linear (dashed lines) and nonlinear (solid lines) conditions and for increasing coupling 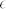. In the simulations we set *σ* = 0.2, ω_0_ = 0.3, *A* = 4 and *K* = 1.8.

We begin our analysis by calculating the momentum diffusion 
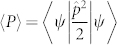
 versus time, with 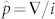 the momentum operator and 〈*ψ*|*f*|*ψ*〉 = *∫ d***r***f*|*ψ*|^2^ the quantum average. In order to investigate a general system, we considered a nonloncal diffusive nonlinear response *n* = *∫ d***r***R*(**r**′ − **r**)|*ψ*(**r**′)|^2^ following from: 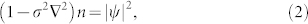
with nonlocality controlled by *σ*. When *σ* = 0, the system response is local with *n* = |*ψ*|^2^. For *σ* ≠ 0, conversely, the system nonlinearity becomes long ranged with kernel given by 
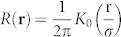
, being *K*_0_ the modified Bessel function of second kind. Diffusive nonlinearities are particularly interesting in the context of nonlinear optics, as they can be easily accessed in liquids, as well as in Bose-Einstein Condensates (BEC), where they generalize previously investigated models^36^,^37^,^38^. Parallel numerical simulations are performed by a direct solution of (1) with an unconditionally stable algorithm. In order for the field *ψ* to explore the periodic potential *U*, we here consider wave packets whose spatial extension Δ*r* ≪ 2*π*. Figure 1 summarizes our results obtained for *σ* = 0.2, by launching at the input a gaussian beam 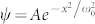 with waist *ω*_0_ = 0.3 and amplitude *A* = 4 (Fig. 1a). The stochastic parameter *K* has been set to *K* = 1.8 > K*, above the stochastization threshold K* ≈ 0.97 where the linear classical uncoupled rotor exhibits diffusive transport in momentum space^22^. For comparison, we also calculated the linear dynamics resulting from *R* = 0 (Fig. 1b dotted line). As seen from Fig. 1b, the 2D nonlinear rotor behaves dramatically different with respect to its linear counterpart, demonstrating the strong role played by nonlinearity in the process. In particular, the linear system exhibits Anderson localization and diffusion suppression for 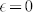 (uncoupled condition), while for growing 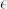 it shows a monotonically increasing sub-diffusion (Fig. 1b). In the nonlinear regime, conversely, Anderson localization is suppressed even for 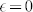, and the dynamics shows an erratic, random-like behavior that does not manifest any simple monotonic increase for growing values of 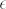. These results are also significantly different from the nonlinear kicked rotor in one dimension^27^, where nonlinearity was observed to induce Anderson localization effects thus suppressing any diffusive (or sub diffusive) regime.

### Analytic theory and Universal diffusion scaling

**Figure 2:**
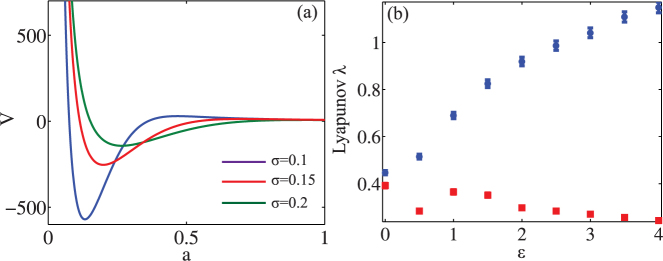
(a) soliton potential 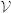 calculated for *P* = 100 and for different non locality degrees *σ*; (b) positive Lyapunov exponent *λ* of the chaotic map (10) versus coupling 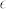, calculated for *K* = 5 and 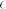 = 0.8.

To theoretically investigate the results of Fig. 1 and derive predictions of universal character, we begin by calculating the time evolution of the wave packet center of mass, thus generalizing the Ehrenfest theorem of classical quantum mechanics: 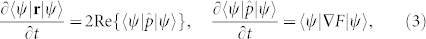
with *F* = *Uδ_T_* + *∫ d***r**′*R*(**r** − **r**′)|*ψ*(**r**′)|^2^. Equations (3) are valid for any dimensionality of the problem and for any nonlinearity *R*. In the following we assume a generic nonlinear response *R* that support at least a stable bound state of minimum energy, i.e., a nonlinear ground state solution. Numerical simulations performed in the previous section showed that the spatial field profile of the wavepacket, despite the chaotic motion, is not significantly altered in time (Fig. 1a,c). We can therefore describe the wavepacket dynamics in terms of a reduced set of coordinates modeling the nonlinear ground state of Eq. (1). To find the general form of the nonlinear ground state, we exploit Lie symmetry groups theory^39^. In particular, we start from the Lagrangian density 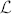 of Eq. (1), written for *U* = 0: 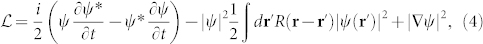
and identify its variational symmetries, which we express by the following basis of Lie generators: 
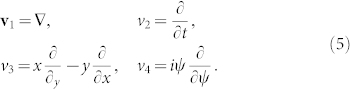
These generators are associated to translational, rotational and Gauge symmetries of (1). The nonlinear ground state of (5) represents an invariant solutions with respect to the global symmetry group generated by: 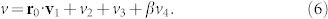
We employed the method of characteristics^39^,^40^ to find the functional form of the general solution, which reads as follows: 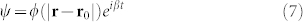
with *ϕ* being a complex envelope. Equation (7) represents a soliton wave of the system. Closed form expression of *ϕ* for integrable responses *R* are found by the inverse scattering transform. In the case of Eq. (2), which possesses a nonintegrable response, we found approximate solutions by a variational analysis^36^,^41^. In particular, we use the following Gaussian ansatz: 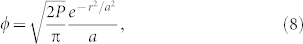
defined by the power *P* = 〈*ψ*|*ψ*〉 and waist *a*(*t*). By substituting Eq. (8) in (4), after long but straightforward algebra we obtain the classical dynamics following from the Hamiltonian 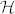: 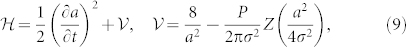
with *Z*(*x*) = *e*^−*x*^Γ(0,*x*), Γ(0,*x*) the upper incomplete gamma function and 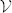 the potential of the one dimensional motion of *a*. The potential 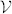 has a bell shape profile that possesses a unique absolute minimum *V*(a*) for every combination of *P* and *σ*. The fixed point *a*(0) = a* corresponds to a soliton wave of the system, which propagates in a translational fashion with fixed waist *a*(*t*) = a*, while different initial values lead to a breather^42^ characterized by a periodic oscillation of *a* in time. Figure 2a shows a typical 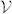 profile obtained for *P* = 100 and different nonlocality degrees *σ*. As seen in the figure, the potential well becomes bigger and bigger for increasing non locality, explaining the dynamical robustness of the soliton dynamics during the chaotic motion observed in the parallel simulations performed in the previous section.

To investigate the motion of the soliton ground state in the general case when *U* ≠ 0 and *R* is arbitrary, we substitute Eq. (7) into (3) and perform an integration from *nT* to (*n* + 1)*T*. After some algebra, we end with the following system: 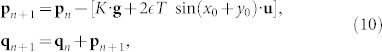
with **g** = [sin *x*_0_, sin *y*_0_], **u** = [1, 1], classical position **q***_n_* ≡ **q**(*nT*) and momentum **p***_n_* ≡ **p**(*nT*) defined from **q** = 〈*ψ*|**r**_0_|*ψ*〉/〈*ψ*|*ψ*〉 and 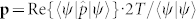, respectively. In the derivation of (10) we assumed the general condition of 〈*ψ*|(**r** − **r**_0_)^2^|*ψ*〉 ≪ 2*π*, in agreement with our introductory premises. Equations (10) represent a two dimensional standard map: for *K* > K*, above the stochastization threshold of the single uncoupled rotor, Eq. (10) is hyperchaotic and each dimension acts as an external noise source to the other, increasing the mixing of the overall system^43^. To highlight such a dynamics, we plot in Fig. 2b the positive Lyapunov exponent *λ* calculated for Eqs. (10). As seen, even when only a single rotor overcomes its stochastization threshold, it provides a noise source to the other giving rise to two positive Lyapunov exponents. The largest Lyapunov exponent grows linearly with 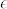 (Fig. 2b). The hyperchaotic nature of Eq. (10) is expect to strongly affect the momentum diffusion in the phase space. We investigate the latter by generalizing the approach of Rechester and White developed for turbulent flows^44^. The classical diffusion *D* of the map can be expressed as follows: 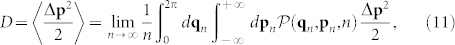
being Δ**p** = **p***_n_* − **p**_0_ and 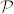 the probability distribution of position **p***_n_* and momentum **q***_n_* at time *n* (measured in kick units). To calculate 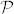, we begin by observing that the the evolution of the map (10) at the *n*-th kick can be written as follows: 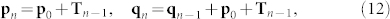
expressed as a function of the initial momentum **p**_0_, with 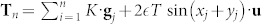 and **g***_j_* = [sin *x_j_*, sin *y_j_*]. The evolution of the probability density 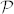 at time *n* is then given by: 
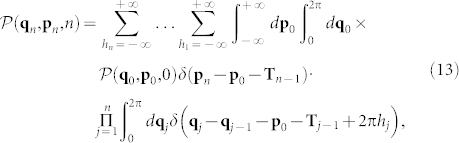
being 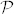
(**q**_0_, **p**_0_,0) the initial density. The presence of the additional summations over *h* originates from the periodicity of the position **q** in the phase space. By considering a general uniform initial distribution with all the particles possessing nonzero momentum **p**, i.e., 
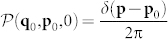
, after substituting into (13) and (15), we obtain: 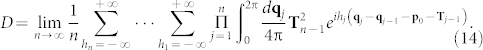
The leading order in the integral is obtained by neglecting any folding in the position space, i.e., by assuming *h_j_* = 0 (for *h_j_* ≠ 0, conversely, we get asymptotically small corrections expressed in terms of Bessel functions that we do not reported here due to their cumbersome expressions). The leading diffusion reads as follows: 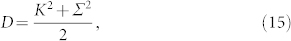
being 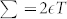. Equation (15) is to be considered of universal applicability, i.e., for any system dimension and for arbitrary nonlinear responses, as it has been derived under the general equations (3), (10)–(15).

## Discussion

**Figure 3:**
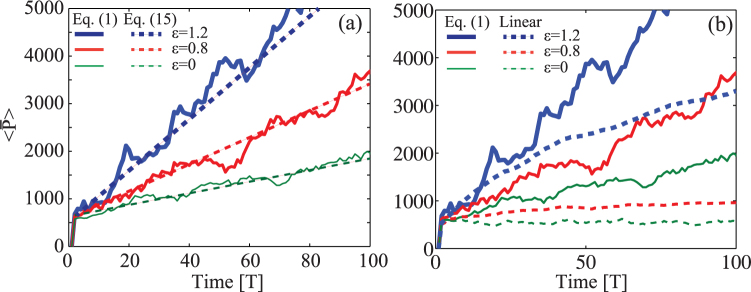
(a) Momentum diffusion 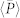 versus time calculated from Eq. (1) (solid lines) and Eqs. (15) (dashed lines). Panel (b) compares the nonlinear diffusive dynamics based on Eq. (1) to the linear transport dynamics obtained in the case of *R* = 0.

Equation (15) allows to fully interpret the nonlinear dynamics of Eq. (1). In particular, the quantum average 〈*P*〉 results from an hyperchaotic system described by a two dimensional dimensional standard map, and each realization manifests itself as a random walk in Fig. 1b. The map diffusion rate is identical to the momentum diffusion of the classical linear rotor^22^, hence, an additional average (in time or over an ensemble of input conditions) re-establishes a perfect classical correspondence for every coupling 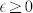. It is worthwhile observing that the classical correspondence in the multidimensional linear quantum rotor is manifested only for very high coupling 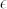, and in general the quantum diffusion 〈*P*〉 follows a fractional behavior with 〈*P*〉 ∝ *t^β^*^<1^ (see e.g.^22^, or Fig. 1b dashed lines). As a result, the linear quantum rotor sub-diffuses at a slower rate than its classical counterpart. Conversely, Eq. (15) predicts a perfect classical correspondence for every coupling 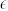, which is re-established thanks to nonlinear effects. In order to verify Eq. (15), and to demonstrate such a nonlinearly-enhanced transport dynamics, we performed extensive numerical simulations from Eq. (1) and calculated the average diffusion through a quantum average followed by an average over different input conditions 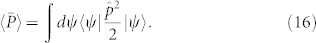
Figure 3 summarizes our results obtained for *K* = 5, *σ* = 0.2 and by considering an initial wave packet composed by a Gaussian beam with waist *ω*_0_ = 0.3 and amplitude *A* = 4. In complete agreement with Eqs. (10)–(15), we observe a diffusive behavior 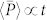 for every 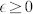 (Fig. 3a solid lines), whose rate is exactly matching our theoretical prediction based on Eq. (15) [Fig. 3a dashed lines]. Figure 3b compares linear and nonlinear evolution of Eq. (1), the former obtained for *R* = 0. As seen in the figure, the nonlinear enhancement in the diffusion due to the restoration of classical effect is significant, and it increases with the coupling 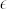. This can be intuitively expected due to the fact that an increasing coupling leads to an increasing mixing in the system and to stronger diffusion effects. The relative diffusion variation 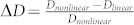, normalized respect to the nonlinear dynamics, is approximatively 50% at 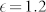, which means that nonlinearity is significantly faster (by a factor of two) in transporting energy.

In order to discuss a possible experimental realization, and further verify the universality of our predictions, we considered the case of a multidimensional dipolar BEC^41^. This system attracted a conspicuous interest in the scientific community due to its important implications in many-body dynamics, quantum computing and nonlinear waves^28^,^45^,^46^,^47^,^48^. Dipolar BEC are characterized by long range interactions, which support stable ground state solitons and high order azimutons^28^,^48^. The nonlinear dynamics of the wavefunction in a kicked optical lattice can be written in the following adimensional form: 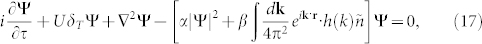
with dimensionless *τ* = *tω_z_*, 
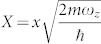
, 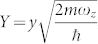, 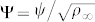, 
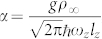
, 
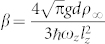
, 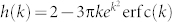, where 
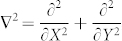
, 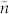 is the Fourier transform of |*Ψ*|^2^, *ω_z_* is the BEC trapping frequency along *z*, 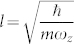, *g* the coupling constant characterizing two-body contact interactions, *g_d_* the strength of the dipole-dipole interaction potential^28^ and *ρ*_∞_ a reference BEC density. Interaction terms *g* and *g_d_* can be tuned via Feshbach resonance, which allows to experimentally control the ratio *g_d_*/*g* that determines the properties of the nonlinear ground state of the condensate. In our numerical experiments, we considered *g_d_*/*g* = −0.5, as a feasible value for the generation of a stable two-dimensional soliton state^28^.

**Figure 4:**
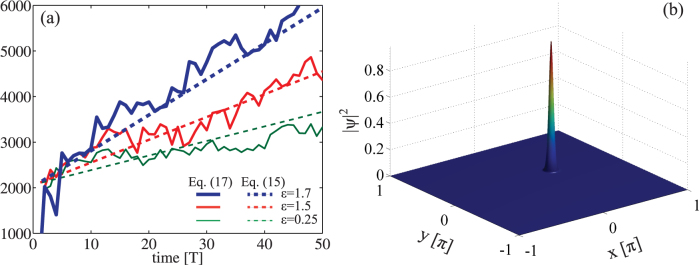
(a) Momentum diffusion 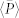 versus time calculated for a dipolar BEC in a kicked optical lattice (solid lines) and theoretical prediction based on the universal Eq. (15) (dashed lines). Panel (b) illustrate the spatial density probability distribution |Ψ|^2^ at time *τ* = 50*T*.

Figure 4 summarizes our results for *K* = 2, *T* = 1 and different coupling strengths 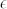. In perfect agreement with our theory, the averaged momentum diffuses as predicted by our universal formula, increasing its strength as the coupling 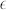 grows. The final density distribution, after *τ* = 50*T*, shows the stable propagation of the dipolar ground state that is evolving inside the system (Fig. 4b), proving the feasibility of our predictions in a realistic system.

In conclusion, motivated by the large interest in the study of the energy transport in disordered system, we investigated the role of nonlinearity in affecting the dynamics of energy diffusion in random media. This problem has also profound implications in quantum chaos and time reversibility of classical systems, where nonlinearity emerges naturally when we consider interacting particles. We considered a universal model of wave propagation, namely the multidimensional quantum kicked rotor in presence of a generic atom-atom interactions. We began our analysis with a single numerical experiment, and studied the behavior of the energy diffusion in the phase space, finding unexpected results that are very different with respect to both linear multidimensional dynamics and nonlinear evolutions of one dimensional systems. We tackled the problem by employing a combination of soliton theory and Lie symmetry analysis, finding a universal diffusion evolution of the energy that follows from the full restoration of classical effects sustained by soliton waves. The latter, in particular, breaks Anderson localization effects and diffuse energy with a larger rate with respect to linear systems of the same size. Numerical simulations performed on different models, including dipolar BEC, perfectly agree with our universal predictions. From a pure quantum perspective, our results demonstrate that atom-atom interactions inhibit quantum time reversal in many dimensions, due to the full recovery of classical chaotic mixing in the system. This generalizes the intuition of Adachi, Today and Ikeda^22^, who found that in linear regime multidimensional quantum time reversal is only *conditionally* possible, in the sense that an 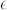 threshold exists for the recovery of the initial wavepacket, while beyond a specific interaction 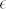 no reversal is possible. When nonlinearity is taken into account, conversely, solitons reestablish a fully mixing dynamics and for all values of 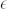 no time reversal can be observed.

We can therefore conclude that nonlinear waves can favor the energy transport in a disordered medium, and significantly speed up the process of energy diffusion when compared to linear dynamics. Solitons can be therefore used for the development of new architectures for enhancing the transport of matter in disordered materials.

## Methods

Numerical simulations of Eq. (1) have been realized by an homemade parallel code based on an unconditionally stable, second order time marching scheme. Parallelization is achieved by a two dimensional domain decomposition strategy, where each part of the computational domain is assigned to a different processor, with all communications written following the *MPI* standard. The numerical results presented in this work have been realized by 200000 single cpu hours on 128 processors of our “reddragon” linux cluster.

## Author Contributions

A.F. conceived the work and developed the theoretical analysis. D.S.B. performed the numerical simulations. A.F. wrote the manuscript. All authors reviewed the manuscript.
